# Characteristics of striatal changes in diabetic retinopathy and type 2 diabetes: a resting-state functional magnetic resonance imaging study

**DOI:** 10.3389/fnhum.2026.1765793

**Published:** 2026-04-28

**Authors:** Man Song, Siyi Lai, Shimei Wei, Liangyan Yi, Duan Wang, Zefeng Kang, Guanghui Liu

**Affiliations:** 1 Department of Ophthalmology, Fuzhou Hospital of Traditional Chinese Medicine Affiliated to Fujian University of Traditional Chinese Medicine, Fuzhou, Fujian, China; 2 Department of Ophthalmology, Eye Hospital China Academy of Chinese Medical Sciences, Beijing, China; 3 Department of Ophthalmology, Affiliated People's Hospital of Fujian University of Traditional Chinese Medicine, Fuzhou, Fujian, China

**Keywords:** diabetic retinopathy, functional connectivity, resting-state fMRI, striatum, type 2 diabetes mellitus

## Abstract

The presence of reward-related neural deficits in type 2 diabetes mellitus (T2DM) and its microvascular complications has been linked to abnormal striatal function. However, differences in striatal whole-brain functional connectivity (FC) between patients with diabetic retinopathy (DR) and those with T2DM without retinopathy remain unclear. In this study, 30 patients with DR, 30 with T2DM, and 29 healthy controls (HCs) were recruited. A seed-based FC approach was employed to analyze alterations across six predefined striatal subregions among the three groups and to examine correlations between abnormal FC patterns and clinical cognitive performance. Results revealed that, compared with the T2DM group, the DR group exhibited reduced FC between several striatal subregions and key nodes within the salience, cognitive control, visual, reward, and sensorimotor networks. Notably, in the DR group, MoCA scores showed a positive correlation with FC between the right dorsal rostral putamen and the left insula. These findings provide novel evidence for distinct striatal circuit alterations in DR and may contribute to identifying neuroimaging markers for its early detection and intervention.

## 1 Introduction

Type 2 diabetes mellitus (T2DM) is a metabolic disorder characterized by disturbances in carbohydrate, lipid, and protein metabolism, with impaired glucose regulation as its core manifestation (Strati et al., 2024). This disease can trigger multiple complications affecting various organs including the kidneys, cardiovascular system, nervous system, and eyes (Kwok et al., 2024). Among these, diabetic retinopathy (DR) stands as one of the most common and severe microvascular complications of T2DM (Hendrick et al., 2015). DR fundamentally represents a circulatory disorder of the retina, impeding the delivery of nutrients essential for its highly metabolic processes. Its progression follows a typical sequence, advancing from mild non-proliferative diabetic retinopathy (NPDR) to moderate-to-severe NPDR, and ultimately reaching proliferative diabetic retinopathy (Kour et al., 2024). PDR is characterized by the formation of fragile, easily ruptured abnormal neovascularization on the retinal surface. These vessels predispose patients to vitreous hemorrhage and traction retinal detachment, representing a high-risk stage for vision loss (Ghamdi, 2020). Concurrently, vascular leakage at any stage of DR can trigger exudate accumulation and retinal edema (Yue et al., 2022). When lesions involve the fovea centralis, diabetic macular edema develops, causing rapid deterioration of central vision (Tatsumi, 2023).

Although DR has a high overall prevalence among diabetic patients, only approximately 5%−10% progress to vision-threatening advanced stages such as PDR or DME (Wong et al., 2006). However, the risk of developing DR varies by diabetes type: the lifetime risk for T2DM patients is approximately 50%−60%, while for type 1 diabetes patients—who typically have longer disease duration and earlier onset—the risk can reach up to 90% (Ghamdi, 2020; Cheung et al., 2022). Therefore, deepening our understanding of DR's pathogenesis is crucial for its effective prevention and treatment.

In recent years, resting-state functional magnetic resonance imaging (rs-fMRI) studies have deepened our understanding of the neuropathological mechanisms underlying T2DM (Dai et al., 2024). Notably, research focusing on the neuropathological differences between T2DM and DR has provided crucial insights into the unique pathogenesis and therapeutic directions for DR (Wang et al., 2017). Functional connectivity (FC), a commonly employed rs-fMRI methodology, analyzes temporal correlations between voxels in regions of interest and distant brain areas to reveal coordination and interactions among neural activities across different brain regions (Cole et al., 2010). Previous studies have demonstrated FC abnormalities in T2DM patients within the default mode network (DMN), affective network (AN), and cognitive control network (CCN; Meng et al., 2022; Antal et al., 2022). However, little is currently known about functional differences between DR and T2DM in reward circuitry.

In patients with T2DM, anhedonia is a relatively common non-cognitive neurological symptom, typically more pronounced in those with central complications such as cerebral microvascular disease or cognitive impairment (Duarte-Silva et al., 2021; Kim et al., 2024). Anhedonia is closely associated with dysfunction in the brain's reward circuitry. The striatum, as a key component of the reward system, participates in integrating an individual's reward evaluation, motivation formation, and behavioral decision-making processes (Castro et al., 2021). Existing evidence suggests that modulating dopamine pathways may help alleviate motivation deficits associated with diabetes, and the functional integrity of the striatum directly influences the efficient transmission of dopamine signaling (Sun et al., 2023). Furthermore, as a pivotal node connecting higher cortical and subcortical structures, the striatum forms part of the cortico-striatal-pallidal-thalamic closed loop, playing an integrative role in metabolic regulation, reward experience, and executive function (Friedman, 1994; Volkow et al., 2011). The striatum exhibits distinct functional subdivisions, primarily comprising the putamen, caudate nucleus and ventral striatum (VS; Wang et al., 2018). Specifically, the ventral striatum receives neural inputs from brain regions involved in emotion and value assessment, such as the orbitofrontal cortex (OFC). The dorsal caudate (DC) primarily receives projections from areas responsible for cognitive control and planning functions, such as the dorsolateral prefrontal cortex. The putamen, meanwhile, is more focused on integrating sensorimotor information (Sun et al., 2023; Wang et al., 2018, 2020). Given its intricate anatomical connections and distinct functional differentiation, the striatum is considered a critical research target for investigating abnormalities in the reward-motivation network associated with T2DM and the underlying mechanisms linking these abnormalities to metabolic dysregulation. Crucially, there is a robust theoretical basis for linking retinal microvascular pathology to striatal circuit dysfunction in DR. First, from a pathological perspective, the retina and the striatum share common embryological origins and similar microvascular physiological environments. The striatum, being a highly metabolically active subcortical nucleus, is particularly vulnerable to the systemic microvascular damage and chronic hyperglycemia characteristic of T2DM (Chithrapathra et al., 2023; Nilofar et al., 2024). Therefore, retinal microvascular lesions in DR may serve as a visible “window,” reflecting parallel microangiopathic changes in the striatum. Second, from a functional network perspective, the striatum is not an isolated reward processor but acts as a critical hub for the “visual-striatal loop” (Peters et al., 2021). It receives extensive projections from the visual cortex to support visual-guided habit formation and reward prediction. The chronic visual impairment or “visual deprivation” caused by DR may disrupt these afferent signals, leading to trans-synaptic degeneration or maladaptive functional reorganization within striatal subregions (Meng et al., 2022). Consequently, investigating striatal functional connectivity in DR patients allows us to capture the neuroplastic changes driven by the dual burden of metabolic-vascular damage and sensory deprivation.

Therefore, this study employed FC analysis to examine differences in brain functional activity between DR and T2DM across distinct subregions of the striatum. It further explored correlations between FC patterns and clinical manifestations. This research contributes to identifying neuroimaging markers at different stages of T2DM progression, while also providing reference for elucidating the pathogenesis of DR and informing clinical diagnosis and treatment.

## 2 Methods

### 2.1 Participants

All subjects in this study were recruited from Fuzhou Hospital of Traditional Chinese Medicine Affiliated to Fujian University of Traditional Chinese Medicine. Diabetes diagnosis followed the World Health Organization (WHO) 1999 criteria for type 2 diabetes mellitus (T2DM; Alberti and Zimmet, 1998). DR diagnosis was based on characteristic fundus lesions resulting from diabetic vascular damage to the retina (Chong et al., 2024). The study included 30 patients with DR and 31 patients with T2DM. Inclusion criteria were as follows (Wang et al., 2017; Yan and Wang, 2023): (1) confirmed DR at stages I–III of simple diabetic retinopathy; (2) age 18–70 years; (3) right-handedness; (4) consciousness intact with ability to express sensations and cognition normally; (5) consistent systemic medication regimen including insulin and hypoglycemic agents; (6) complete clinical records.

Meanwhile, 29 age- and gender-matched healthy controls (HCs) were recruited. Inclusion criteria for the healthy control group were: (1) age 18–70 years; (2) right-handedness; (3) complete clinical records.

Exclusion criteria for all subjects were: (1) presence of MRI scanning contraindications; (2) other concomitant ocular diseases that could affect the visual system or confound the diagnosis of DR, including glaucoma, significant cataracts, age-related macular degeneration, retinal detachment, optic neuritis, and high myopia; (3) psychiatric disorders; (4) pregnancy or lactation; (5) history of alcohol dependence.

### 2.2 Clinical scale assessment

All participants underwent the Mini-Mental State Examination (MMSE) and the Montreal Cognitive Assessment (MoCA) to evaluate cognitive function prior to completing rs-fMRI scans.

### 2.3 Scan acquisition

All subjects in this study underwent rs-fMRI data acquisition at the Radiology Department of Fuzhou Hospital of Traditional Chinese Medicine Affiliated to Fujian University of Traditional Chinese Medicine. The imaging was performed using a Siemens Magnetom Skyra 1.5T scanner (Germany). Prior to scanning, subjects' heads were secured with a headrest, and they were instructed to wear noise-canceling headphones while lying supine on the examination table. Subjects were required to remain awake and relaxed throughout the procedure.

Scanning parameters were as follows: Three-dimensional T1-weighted structural images used the following settings: Repetition time/Echo time (TR/TE) = 1,900/2.48 ms, Matrix = 64 × 64, Flip angle = 90 °, Slice thickness = 1 mm, Field of view = 256 mm × 256 mm, Number of slices = 176, Scan duration = 5 min 50 s. Blood oxygen level-dependent functional imaging (BOLD-fMRI) scan parameters: TR/TE: 2,000/25 ms, matrix 64 × 64, number of slices 43, flip angle 90 °, field of view 240 mm × 240 mm, scan duration 6 min 45 s.

### 2.4 Image processing

#### 2.4.1 fMRI data preprocessing

Functional magnetic resonance imaging data underwent preprocessing via the Data Processing Assistant for Resting-State fMRI (DPARSF) software (version 6.0, accessible at http://www.rfmri.org/DPARSF), which is based on Statistical Parametric Mapping 12 (SPM12, http://www.fil.ion.ucl.ac.uk/spm) running in MATLAB (Kroemer et al., 2022). The preprocessing pipeline comprised the following steps: (1) conversion of raw DICOM images to the NIFTI format; (2) exclusion of the initial ten time points from the NIFTI dataset; (3) slice timing correction; (4) realignment for head motion, with participants exhibiting translational movement exceeding 2 mm or rotational displacement greater than 2 degrees being excluded; (5) coregistration of the realigned functional time-series for each participant with their corresponding high-resolution T1-weighted anatomical image, followed by spatial normalization to the Montreal Neurological Institute standard space using the DARTEL algorithm, resampled at an isotropic resolution of 3 mm^3^; (6) linear detrending to attenuate scanner-related low-frequency drift; (7) nuisance covariate regression, incorporating signals from white matter, cerebrospinal fluid, and estimated motion parameters; (8) spatial smoothing with an isotropic Gaussian kernel of 6 mm full-width at half-maximum; and (9) temporal band-pass filtering within the 0.01–0.08 Hz frequency range.

#### 2.4.2 Seed-based functional connectivity

Striatal subregion seed point coordinates were defined according to established literature (Sun et al., 2023; Wang et al., 2018, 2020). The MNI 152 standard space coordinates for each region were as follows: inferior ventral striatum (VSi) at (x = ±9, y = 9, z = −8), superior ventral striatum (VSs) at (x = ±10, y = 15, z = 0), DC at (x = ±13, y = 15, z = 9), dorsal caudal putamen (DCP) at (x = ±28, y = 1, z = 3), dorsal rostral putamen (DRP) at (x = ±25, y = 8, z = 6), and ventral rostral putamen (VRP) at (x = ±20, y = 12, z = −3). Each spherical seed region encompassed 27 voxels (radius = 4 mm) in 2-mm^3^ isotropic space. For every participant, the average time series within each region of interest (ROI) was computed. Whole-brain functional connectivity maps were subsequently generated by calculating Pearson's correlation coefficients between each ROI time series and the time series of all brain voxels. These correlation coefficients underwent Fisher's Z-transformation to approximate a normal distribution for further statistical analysis.

### 2.5 Statistical analyses

#### 2.5.1 Clinical data analysis

Statistical analysis of the clinical data was performed with SPSS 26.0 (IBM Corp, Armonk, NY, USA). Group comparisons for sex distribution were conducted using the chi-square test. Differences across the three groups in age, years of education, fasting glucose, and HbA1c levels were assessed via one-way analysis of variance (ANOVA). MoCA scores, MMSE scores, and disease duration were compared between the two patient groups using independent two-sample *t*-tests. A significance threshold of *P* < 0.05 was applied for all statistical tests.

#### 2.5.2 fMRI data analysis

Statistical analysis of imaging data was performed using DPARSF 6.0. Whole-brain FC differences across the three groups were examined with one-way ANOVA, while controlling for age, sex, education, and mean framewise displacement (FD; from Jenkinson's formula < 0.2). Corrections for multiple comparisons were applied using Gaussian random field (GRF) theory, with a cluster-level threshold of (*P* < 0.05; two-tailed) and a voxel-level threshold of (*P* < 0.001). Meanwhile, clusters comprising less than 20 voxels were removed.

Mean FC values of identified abnormal brain regions were extracted for each group using DPARSF. Between-group comparisons were conducted in SPSS 26.0 using two-sample *t*-tests, applying Bonferroni correction with a significance level of (*P* < 0.016; 0.05/3).

Pearson correlation analyses were performed between FC values in abnormal regions and cognitive scores (MoCA and MMSE) in the DR and T2DM groups, controlling for age, sex, education, and FD values. Statistical significance was set at (*P* < 0.05; two-tailed).

## 3 Results

### 3.1 Characteristics of research samples

In this study, one patient with T2DM was excluded due to excessive FD. Thus, 30 patients with DR, 30 patients with T2DM without retinopathy, and 29 HCs were included in the final analysis. The three groups did not differ significantly in age, sex, or years of education. An independent-samples *t*-test indicated no statistically significant difference in disease duration between the DR and T2DM groups. One-way analysis of variance (ANOVA) revealed significant differences across the three groups in fasting glucose, HbA1c, MoCA scores, and MMSE scores. However, *post-hoc* two-sample *t*-tests demonstrated no statistically significant differences in fasting glucose, HbA1c, MoCA scores, and MMSE scores between the DR and T2DM groups (Table 1).

**Table 1:** Demographic and clinical characteristics of the study participants.

Characteristics	DR group	T2DM group	HC group	*F*/*t*/χ^2^	*p*-value
Age (years)	54.20 ± 7.37	55.16 ± 7.70	54.50 ± 7.12	0.134	0.875^a^
Sex (F/M)	16/14	18/12	16/14	0.360	0.835^b^
Education (years)	14.60 ± 3.31	14.40 ± 3.12	14.43 ± 3.23	0.033	0.967^a^
Duration of disease (years)	11.13 ± 6.07	10.93 ± 5.54	NA	0.133	0.895^c^
Fasting glucose (mmol/l)	8.80 ± 1.56	8.70 ± 1.29	5.23 ± 0.67	107.15	< 0.001^a*^
HbA1c (%)	8.66 ± 1.54	8.50 ± 1.38	5.57 ± 0.45	78.953	< 0.001^a*^
MoCA	21.66 ± 1.17	21.80 ± 1.29	25.10 ± 1.18	77.029	< 0.001^a*^
MMSE	27.63 ± 0.49	27.73 ± 0.44	28.56 ± 0.89	18.933	< 0.001^a*^

DR, diabetic retinopathy; T2DM, type II diabetes mellitus; HCs, healthy controls; NA, not applicable.

^a^One-way analysis of variance was employed for comparisons among the three groups. ^b^The chi-square test was applied to compare sex distribution across the groups. ^c^A two-sample t-test was conducted for the comparison between two groups.

^*^Indicates statistical significance.

### 3.2 Among the three group differences in striatal FC

#### 3.2.1 VSi, VSs

**Figure 1:**
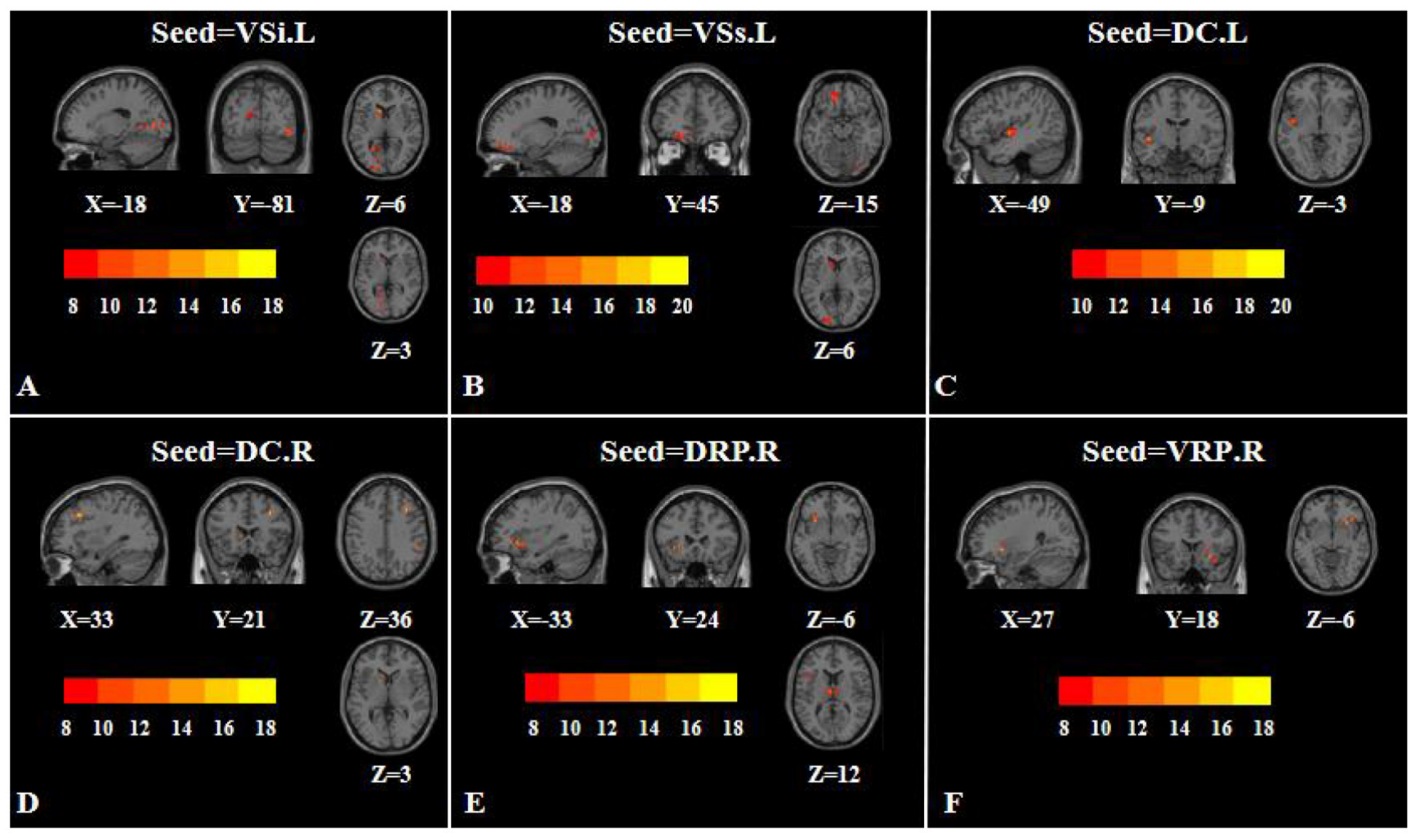
Group differences in striatal FC based on one-way analysis of variance. **(A)** Seed = left inferior ventral striatum (VSi.L); **(B)** Seed = left superior ventral striatum (VSs.L); **(C)** Seed = left dorsal caudate (DC.L); **(D)** Seed = right dorsal caudate (DC.R); **(E)** Seed = right dorsal rostral putamen (DRP.R); **(F)** Seed = right ventral rostral putamen (VRP.R). The color bars represent F-values. L, left; R, right; VSi, inferior ventral striatum; VSs, superior ventral striatum; DC, dorsal caudate; DRP, dorsal rostral putamen; VRP, ventral rostral putamen.

One-way ANOVA revealed that, with the left VSi as the seed, significant group differences were found in the left lingual gyrus, right inferior occipital gyrus, and left caudate (Table 2; Figure 1A). With the left VSs as the seed, significant differences were observed in the left orbital superior frontal gyrus, left superior occipital gyrus, and left caudate (Table 2; Figure 1B).

**Table 2:** Group differences in striatal FC.

Clusters	Brain regions	MNI peak			Cluster size	*F*-value (peak)
		X	Y	Z		
VSi.L						
1	Lingual_L	−18	−75	3	58	15.230
2	Occipital_Inf_R	39	−81	−9	25	14.079
3	Caudate_L	−12	18	6	25	18.433
VSs.L						
1	Frontal_Sup_Orb_L	−18	45	−15	68	15.704
2	Occipital_Sup_L	−15	−93	6	58	13.829
3	Caudate_L	−9	6	9	27	15.059
DC.L						
1	Insula_L	−49	−9	−3	34	18.393
DC.R						
1	SupraMarginal_R	48	−42	36	25	12.211
2	Frontal_Mid_R	33	21	36	22	19.391
3	Caudate_L	−9	21	3	21	13.391
DRP.R						
1	Insula_L	−33	24	−6	45	18.731
2	Thalamus_L	−6	−12	12	39	13.902
VRP.R						
1	Putamen_R	27	18	−6	53	19.467

Primary peak locations are reported in Montreal Neurological Institute (MNI) coordinates.

#### 3.2.2 DC

One-way ANOVA revealed that, with the left DC as the seed, significant group differences were found in the left insula (Table 2; Figure 1C). With the right DC as the seed, significant differences were observed in the right supramarginal gyrus, right middle frontal gyrus, and left caudate (Table 2; Figure 1D).

#### 3.2.3 DRP, VRP

One-way ANOVA revealed that, with the right DRP as the seed, significant group differences were found in the left insula and left thalamus (Table 2; Figure 1E). With the right VRP as the seed, significant differences were observed in the right putamen (Table 2; Figure 1F).

### 3.3 *Post-hoc t*-test analysis of intergroup differences in striatal FC

#### 3.3.1 VSi, VSs

**Figure 2:**
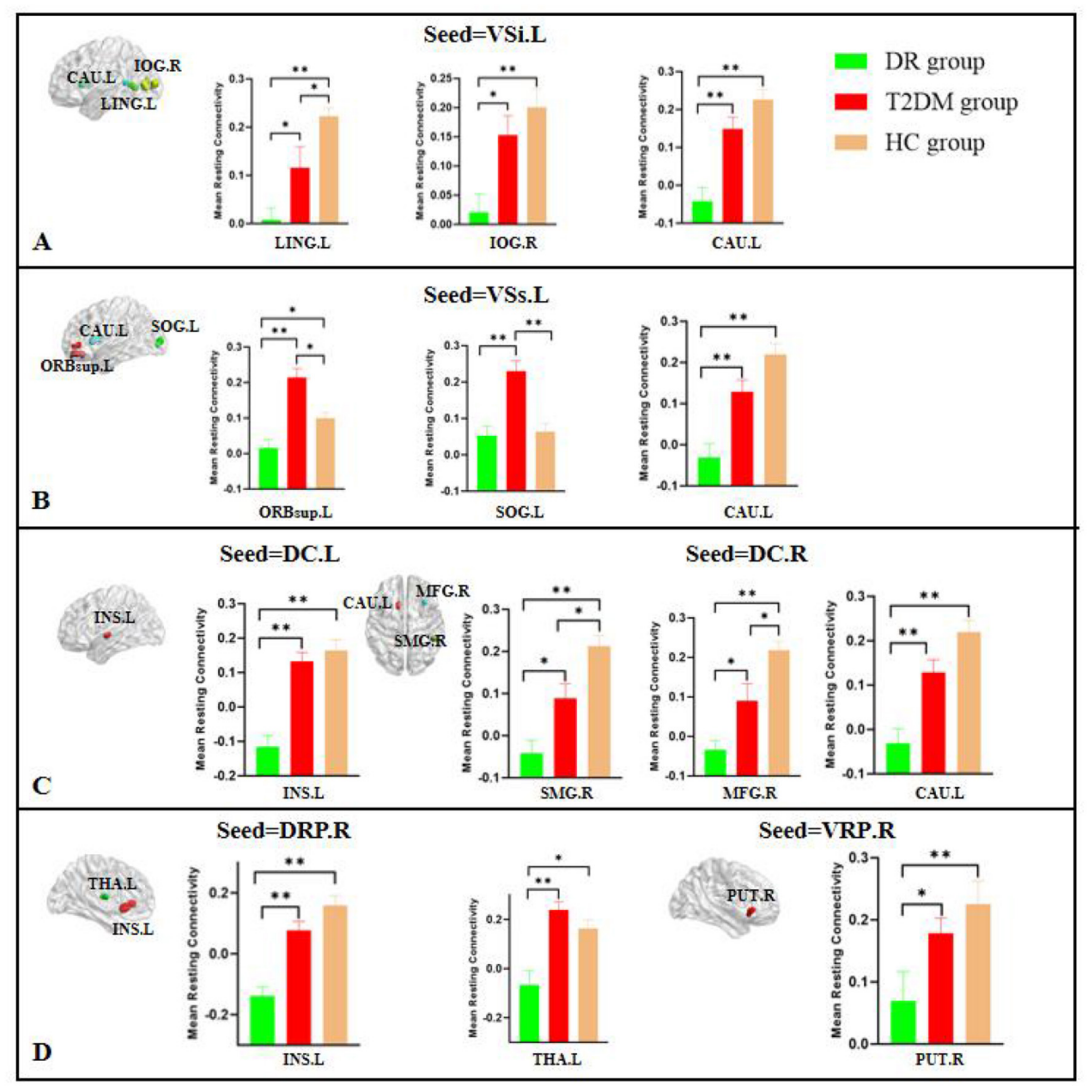
Bonferroni-corrected *post-hoc* tests revealed differences in FC values at the peak voxel between each pair of groups (DR group vs. T2DM group, DR group vs. HC group, T2DM group vs. HC group). **(A)** Seed = left inferior ventral striatum (VSi.L), showing FC differences in LING.L, IOG.R, and CAU.L; **(B)** Seed = left superior ventral striatum (VSs.L), showing FC differences in ORBsup.L, SOG.L, and CAU.L; **(C)** Seed = left dorsal caudate (DC.L) and right dorsal caudate (DC.R), showing FC differences in INS.L, SMG.R, MFG.R, and CAU.L; **(D)** Seed = right dorsal rostral putamen (DRP.R) and right ventral rostral putamen (VRP.R), showing FC differences in INS.L, THA.L, and PUT.R. LING, lingual gyrus; IOG, inferior occipital gyrus; CAU, caudate; ORBsup, orbital superior frontal gyrus; SOG, superior occipital gyrus; INS, insula; SMG, supramarginal gyrus; MFG, middle frontal gyrus; THA, thalamus; PUT, putamen. ^**^P < 0.001; ^*^P < 0.016; L, left; R, right.

Compared with the T2DM group, the FC of the left VSi with the left lingual gyrus, right inferior occipital gyrus, and left caudate were reduced in the DR group. Compared with the HC group, the FC of the left VSi with the left lingual gyrus, right inferior occipital gyrus, and left caudate were reduced in the DR group, and the FC of the left VSi with the left lingual gyrus was reduced in the T2DM group (Figure 2A).

Compared with the T2DM group, the FC of the left VSs with left orbital superior frontal gyrus, left superior occipital gyrus, and left caudate were reduced in the DR group. Compared with the HC group, the FC of the left VSs with the left orbital superior frontal gyrus and left caudate were reduced in the DR group, and the FC of the left VSs with the left orbital superior frontal gyrus and left superior occipital gyrus were reduced in the T2DM group (Figure 2B).

#### 3.3.2 DC

Compared with the T2DM group, the FC of the left DC with the left insula was reduced in the DR group. Compared with the HC group, the FC of the left DC with the left insula was reduced in the DR group (Figure 2C).

Compared with the T2DM group, the FC of the right DC with the right supramarginal gyrus, right middle frontal gyrus, and left caudate were reduced in the DR group. Compared with the HC group, the FC of the right DC with the the right supramarginal gyrus, right middle frontal gyrus, and left caudate were reduced in the DR group, and the FC of the right DC with the the right supramarginal gyrus and right middle frontal gyrus were reduced in the T2DM group (Figure 2C).

#### 3.3.3 DRP, VRP

Compared with the T2DM group, the FC of the right DRP with the left insula and left thalamus were reduced in the DR group. Compared with the HC group, the FC of the right DRP with the left insula and left thalamus were reduced in the DR group (Figure 2D).

Compared with the T2DM group, the FC of the right VRP with the right putamen was reduced in the DR group. Compared with the HC group, the FC of the right VRP with the the right putamen was reduced in the DR group (Figure 2D).

### 3.4 Relationship between FC and clinical features

**Figure 3:**
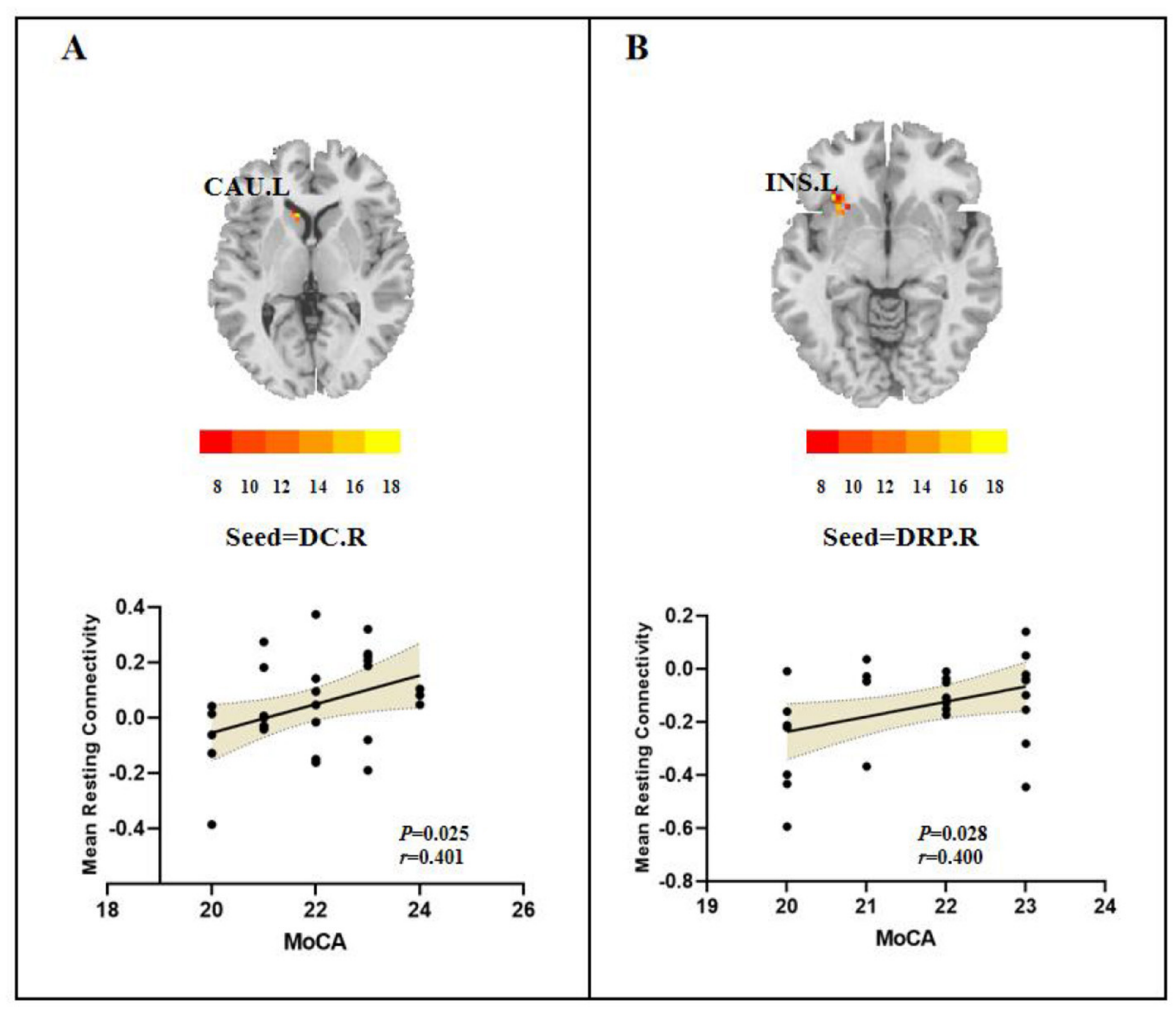
Relationship between FC and clinical features. **(A)** FC values in the T2DM group. **(B)** FC values in the DR group. CAU.L, left caudate; INS.L, left insula; MoCA, Montreal cognitive assessment.

Pearson correlation analyses, controlling for age, sex, education, and FD, were conducted to assess the relationship between FC in striatal subregions and clinical symptoms within each group. In the T2DM group, MoCA scores positively correlated with FC between the right DC and left caudate (*r* = 0.401, *P* = 0.025; Figure 3A), whereas in the DR group, the MoCA scores were positively correlated with the FC between the right DRP and the left insula (*r* = 0.400, *P* = 0.028; Figure 3B).

## 4 Discussion

To our knowledge, this study is the first to investigate differences in striatal circuit FC between DR and T2DM patients. Results revealed significant differences in striatal circuit FC between the two patient groups. Abnormal FC was primarily distributed across key regions of the following brain networks, including the insular in the salience network (SN), the middle frontal gyrus in the CCN, the lingual gyrus and occipital lobe in the visual network (VN), the caudate nucleus, OFC, and thalamus in the reward network (RN), and the parietal lobe in the sensorimotor network (SMN). Furthermore, compared with HCs, both patient groups exhibited partial abnormal changes in FC across multiple striatal subregions, including the VSi, VSs, DC, DRP, and VRP. Notably, in the DR group, abnormal FC between the right DRP and left insula positively predicted cognitive function.

The results of this study indicate that compared with the T2DM group, the DR group exhibited reduced FC between the left DC and the left insula, as well as reduced FC between the right DRP and the left insula. The insula serves as a critical hub within the SN, playing a pivotal role in detecting behaviorally salient stimuli and facilitating the dynamic switching between the DMN and the CCN to support high-order cognitive control (Kroemer et al., 2022; Zhang et al., 2021; Cai et al., 2016). Rather than merely processing interoceptive signals, the insula is integral to maintaining the brain's “cognitive flexibility.” Recent studies suggest that the chronic low-grade inflammatory state associated with DR may disrupt this delicate network efficiency. Peripheral inflammatory cytokines can penetrate the blood-brain barrier and target susceptible hubs like the insula, leading to neuroinflammation-driven network reorganization (Yue et al., 2022; Duarte-Silva et al., 2021). Relevant sensory information is primarily transmitted via the reticular formation and spinal-mesial pathways to the medial thalamus and medial nuclei, subsequently projecting to limbic cortical regions including the anterior insula, OFC, and other prefrontal areas (De Ridder et al., 2022; Rolls et al., 2020). Previous studies have demonstrated reduced FC between the anterior insula and the right inferior frontal gyrus, hippocampus, and precentral gyrus in T2DM patients compared to HCs, consistent with the findings of this study (Zhang et al., 2021). A Granger causality analysis study further revealed that during cognitive control, the anterior insula acts as an information outflow center, regulating posterior parietal cortex involvement in cognitive control (Cai et al., 2016). As cognitive demands increase, activity in the anterior insula and posterior parietal cortex correspondingly enhances, suggesting that cognitive control deficits in T2DM patients may be associated with dysregulation of this mechanism. Additionally, this study found that MoCA scores in the DR group positively correlated with FC between the right dorsal striatum and left insula, suggesting this FC may serve as a potential neuroimaging marker for DR. Thus, this research indicates that weakened FC between striatal subregions and the insula in DR patients may contribute to the pathophysiological mechanisms of DR and holds promise as a key target for future intervention therapies.

Compared with the T2DM group, the DR group exhibited reduced FC between the right DC and the right middle frontal gyrus. Located in the dorsolateral prefrontal cortex, the MFG is a key CCN and plays a crucial role in attention regulation, working memory, and the reorientation of verbal comprehension (Sendi et al., 2023; Wang et al., 2024). Previous studies have indicated that T2DM patients often experience recurrent hypoglycemia due to treatment, a condition closely associated with negative emotional states (Skalkos et al., 2020). Emotion-induction experiments further revealed that emotion-related brain regions, such as the superior frontal gyrus, motor cortex, insula, and inferior frontal gyrus, exhibit heightened sensitivity to blood glucose fluctuations (Kohn et al., 2015). One study indicated that T2DM patients frequently exhibit abnormal FC in the prefrontal cortex-striatal circuit. Specifically, FC strength between the right middle frontal gyrus and right putamen showed a significant positive correlation with fasting blood glucose levels, suggesting that dysfunction in the prefrontal-striatal circuit may be associated with the neuropathological mechanisms of T2DM (Fu et al., 2023). A meta-analysis revealed gray matter reduction in the medial superior frontal gyrus, insula, and anterior cingulate cortex of T2DM patients compared to healthy controls, suggesting gray matter atrophy in the cortex-striatum-limbic network may underlie cognitive impairment in T2DM (Antal et al., 2022). Although our study did not directly measure brain structural metrics, the observed functional disconnectivity in the DR group might be associated with underlying structural impairments reported in previous literature (Liu et al., 2017; Yao et al., 2021). We speculate that the widespread reduction in striatal FC detected in this study could reflect a preliminary stage of “structure-function decoupling,” where functional network efficiency declines prior to or in tandem with gross anatomical damage. These findings suggest that FC abnormalities between the DC and CCN may represent a key neural mechanism underlying DR pathogenesis. Therefore, the present findings suggest that the DR group exhibits more pronounced FC abnormalities between the DC and CCN compared to the T2DM group, which may represent a key neural mechanism underlying DR pathogenesis.

In this study, compared with the T2DM group, the DR group exhibited reduced FC between the left VSi and the left lingual gyrus, as well as between the right occipital subgyrus and the left VSs. The right lingual gyrus, located in the occipital lobe, primarily governs visual memory and visual information processing (Li et al., 2017; Zhang et al., 2024; Wen et al., 2018). Visual information transmitted from the retina is progressively relayed along occipito-temporal and occipito-parietal pathways, entering the occipital visual cortex via the lateral geniculate nucleus of the thalamus for integration and processing (Huang et al., 2019). Previous studies suggest that the DR group exhibits elevated low-frequency amplitude in the right inferior occipital gyrus and left lingual gyrus, indicating that abnormalities in visual processing regions may be associated with the onset and progression of DR. (Wang et al. 2017) reported abnormal brain activity in the occipital region of DR patients, potentially linked to prolonged reduction in visual center stimulation. In their investigation of FC alterations in DR patients, (Qi et al. 2021) hypothesized that chronic visual input reduction may cause decreased visual cortex FC in DR patients, while elevated FC observed in other brain regions could represent compensatory connectivity enhancement triggered by prolonged visual impairment. Collectively, these findings suggest that abnormal functional connectivity between the striatum and visual processing cortex may represent a key neuropathological mechanism distinguishing DR from T2DM.

The OFC, serving as the brain's “decision-making and emotional center,” primarily governs value judgments, impulse control, and social behavior regulation (Brown et al., 2024; Schreiner and Gremel, 2018). The caudate and putamen together form the striatum, which dominates motor programming, reward feedback, and habit formation (Hörtnagl et al., 2020; Brovelli et al., 2011; Karnath et al., 2002). The thalamus serves as a critical information relay station, filtering and integrating sensory signals before they reach the cerebral cortex, while also maintaining conscious awareness (Cassel and Pereira de Vasconcelos, 2021; Pritz, 2025). Together, these three brain regions form the core network underpinning cognition, motor control, and sensory processing, constituting vital components of the RN (Wagner et al., 2023). As a systemic disease, T2DM and its associated cumulative metabolic burden and microvascular complications not only damage the retina but also directly impair the brain, triggering diabetic encephalopathy and cerebral microvascular disease. Importantly, the clinical manifestation of DR may serve as an indicator of an individual's broader systemic vulnerability to microangiopathy. This leads to neuronal degeneration, structural atrophy, or abnormal functional connectivity in brain regions such as the OFC, striatum, and thalamus (Badulescu et al., 2024; Yan et al., 2025). Furthermore, prolonged reduction in visual input can induce “trans-synaptic degeneration,” causing degenerative changes throughout the entire visual pathway from the retina through the thalamus to higher cortical areas. This indirectly affects cognitive and emotional regulation networks dependent on visual input (Liang et al., 2018). Previous studies have shown negative activation in the OFC of T2DM patients, suggesting that functional deficits in this region correlate with impaired decision-making abilities (Sun et al., 2017). Another meta-analysis also indicated gray matter volume reduction in the ventral striatum, putamen, and orbitofrontal cortex of T2DM patients, suggesting that T2DM may accelerate brain aging through neurometabolic mechanisms (Antal et al., 2022). Therefore, the findings of this study suggest that functional abnormalities within the RN of DR patients may represent a key pathogenic mechanism, with these abnormalities being more pronounced than those caused by T2DM.

This study also found reduced FC between the right dorsal cingulate cortex and the right supramarginal gyrus in DR patients compared to HCs. As a higher-order integration hub of the SMN, the supramarginal gyrus primarily integrates visual, somatosensory, and motor information to maintain precise body schema (Hsu et al., 2023; Liu et al., 2019). T2DM-related microvascular complications and neurometabolic abnormalities can directly impair white matter connectivity and gray matter structure in this brain region, thereby weakening sensorimotor integration function (Yao et al., 2021). Previous studies indicate elevated ALFF in the parietal cortex of DR patients, suggesting potential local compensatory functional enhancement in this brain region (Wang et al., 2017). Therefore, this study suggests that abnormal FC between the striatum and SMN in DR patients may represent a key component of their neuropathological mechanisms.

Furthermore, when interpreting the reduced functional connectivity observed in this study, it is crucial to consider the potential confounding role of impaired neurovascular coupling. The BOLD-fMRI signal is an indirect measure of neuronal activity that relies heavily on intact hemodynamic responses (Cole et al., 2010). Diabetic retinopathy is fundamentally a manifestation of systemic microvascular complications (Hendrick et al., 2015), and growing evidence suggests that this microangiopathy likely extends to the cerebral microcirculation (Yuan et al., 2024; Cheung et al., 2015). The striatum, a subcortical hub with high metabolic demand, is particularly vulnerable to such perfusion deficits. Therefore, the widespread reduction in striatal FC observed in the DR group may not solely represent a decline in neuronal synchronization but could also partially reflect “hemodynamic blunting” or neurovascular uncoupling caused by diabetic vascular pathology. While our current rs-fMRI data cannot mathematically disentangle these two factors, we acknowledge that the “functional disconnectivity” reported here is likely a composite outcome of both neuronal degeneration and vascular impairment. Future studies incorporating perfusion imaging techniques, such as Arterial Spin Labeling, are warranted to rigorously clarify the vascular contribution to these functional network alterations.

Notably, although the DR and T2DM groups exhibited comparable fasting glucose and HbA1c levels in the current study (Table 1), the DR group demonstrated more severe and widespread striatal FC deficits. This apparent discrepancy may be attributed to several interrelated mechanisms. First, cross-sectional measurements of HbA1c primarily reflect glycemic control over the preceding 2–3 months, failing to capture historical glycemic fluctuations or the cumulative metabolic burden, often referred to as “metabolic memory,” which is a significant driver of microvascular damage (Ghamdi, 2020; Ceriello, 2012). Second, the manifestation of DR under similar current glycemic conditions suggests a pronounced individual vulnerability to microvascular endothelial dysfunction. Given that the retina and the striatum share common embryological origins and similar microvascular physiological environments, the presence of DR likely signifies concurrent and more severe cerebral microangiopathy (Yuan et al., 2024; Cheung et al., 2015). This innate microvascular fragility could lead to profound neurovascular uncoupling and subsequent functional network disruptions in the DR group. Third, as previously discussed, DR patients endure a dual burden of metabolic-vascular damage and sensory deprivation. Beyond the shared metabolic impairment, the chronic reduction in visual input associated with DR independently induces trans-synaptic degeneration and maladaptive functional reorganization within the visual-striatal loops (Wang et al., 2017; Qi et al., 2021). Therefore, the widespread FC deficits observed in the DR group are likely the synergistic outcome of heightened microvascular susceptibility and visual sensory deprivation, rather than being driven solely by concurrent hyperglycemia.

## 5 Limitations

his study still has several limitations that should be noted. First, the relatively small sample size may affect statistical power and the generalizability of the results. Future studies should expand the sample size and consider including DR subgroups with different disease durations and severity levels for analysis. Second, this study focused only on functional connectivity within the striatum and its subregions, without comprehensively examining other brain networks potentially related to DR, such as the DMN, the limbic system, and others. Future research could extend to whole-brain network analyses to more systematically reveal abnormalities in the neural circuits associated with DR. Third, although this study controlled for covariates such as age, sex, years of education, and head motion, it did not systematically assess patients' depressive or anxiety symptoms, or medication usage—factors that may influence brain functional connectivity. Fourth, and critically, this study relied exclusively on resting-state fMRI data. We did not acquire or analyze multimodal structural data, such as gray matter volume or white matter integrity. Consequently, we could not quantitatively verify whether the observed functional connectivity deficits are caused by “structure-function decoupling” or direct anatomical atrophy. Future studies should integrate multimodal imaging techniques to rigorously test the hypothesis of structure-function decoupling in DR patients. Finally, this study is limited by its reliance on a single imaging modality. Without the incorporation of structural or perfusion imaging, it is difficult to definitively determine whether the observed changes in FC originate from direct neuronal activity alterations or impaired neurovascular coupling. Furthermore, this unimodal approach prevents the validation of the structure-function decoupling hypothesis, leaving it unclear whether functional connectivity decline accompanies or precedes structural atrophy and white matter disruption. Future studies integrating multi-modal neuroimaging are warranted to systematically investigate the underlying structure-function interactions.

## 6 Conclusion

In summary, this rs-fMRI study revealed distinct patterns of striatal functional connectivity alterations in patients with DR compared to those with T2DM without retinopathy and HCs. Specifically, DR patients exhibited widespread reductions in functional connectivity between multiple striatal subregions and key nodes within the SN, CCN, VN, RN, and SMN. These alterations were more pronounced in the DR group relative to the T2DM group, suggesting that DR may involve unique neuropathological mechanisms beyond those associated with T2DM alone. Moreover, the positive correlation between FC of the right DRP with the left insula and cognitive performance in DR patients highlights a potential neuroimaging marker linked to cognitive function in this population. These findings underscore the involvement of striatal circuits in the neural pathophysiology of DR and provide preliminary evidence for its distinction from uncomplicated T2DM at the functional network level.

## Data Availability

The raw data supporting the conclusions of this article will be made available by the authors, without undue reservation.

## References

[B1] Alberti K. G. Zimmet P. Z. (1998). Definition, diagnosis and classification of diabetes mellitus and its complications. Part 1: diagnosis and classification of diabetes mellitus provisional report of a WHO consultation. Diabet. Med. 15, 539–553. doi: 10.1002/(SICI)1096-9136(199807)15:7<539::AID-DIA668>3.0.CO;2-S 9686693

[B2] Antal B. McMahon L. P. Sultan S. F. Lithen A. Wexler D. J. Dickerson B. . (2022). Type 2 diabetes mellitus accelerates brain aging and cognitive decline: complementary findings from UK Biobank and meta-analyses. Elife 11:e73138. doi: 10.7554/eLife.73138 35608247 PMC9132576

[B3] Badulescu S. Tabassum A. Le G. H. Wong S. Phan L. Gill H. . (2024). Glucagon-like peptide 1 agonist and effects on reward behaviour: a systematic review. Physiol Behav. 283:114622. doi: 10.1016/j.physbeh.2024.114622 38945189

[B4] Brovelli A. Nazarian B. Meunier M. Boussaoud D. (2011). Differential roles of caudate nucleus and putamen during instrumental learning. Neuroimage 57, 1580–1590. doi: 10.1016/j.neuroimage.2011.05.059 21664278

[B5] Brown C. S. Devine S. Otto A. R. Bischoff-Grethe A. Wierenga C. E. (2024). Greater reliance on model-free learning in adolescent anorexia nervosa: an examination of dual-system reinforcement learning. medRxiv. doi: 10.1101/2024.01.31.24302097 38352608 PMC10863009

[B6] Cai W. Chen T. Ryali S. Kochalka J. Li C. S. Menon V. (2016). Causal interactions within a frontal-cingulate-parietal network during cognitive control: convergent evidence from a multisite-multitask investigation. Cereb. Cortex. 26, 2140–2153. doi: 10.1093/cercor/bhv046 25778346 PMC4830290

[B7] Cassel J. C. Pereira de Vasconcelos A. (2021). Routes of the thalamus through the history of neuroanatomy. Neurosci. Biobehav. Rev. 125, 442–465. doi: 10.1016/j.neubiorev.2021.03.001 33676963

[B8] Castro D. C. Oswell C. S. Zhang E. T. Pedersen C. E. Piantadosi S. C. Rossi M. A. . (2021). An endogenous opioid circuit determines state-dependent reward consumption. Nature 598, 646–651. doi: 10.1038/s41586-021-04013-0 34646022 PMC8858443

[B9] Ceriello A. (2012). The emerging challenge in diabetes: the “metabolic memory”. Vascul. Pharmacol. 57, 133–138. doi: 10.1016/j.vph.2012.05.005 22609133

[B10] Cheung C. Y. Ikram M. K. Klein R. Wong T. Y. (2015). The clinical implications of recent studies on the structure and function of the retinal microvasculature in diabetes. Diabetologia 58, 871–885. doi: 10.1007/s00125-015-3511-1 25669631

[B11] Cheung N. Chee M. L. Klein R. Klein B. E. K. Shea S. Cotch M. F. . (2022). Incidence and progression of diabetic retinopathy in a multi-ethnic US cohort: the multi-ethnic study of atherosclerosis. Br. J. Ophthalmol. 106, 1264–1268. doi: 10.1136/bjophthalmol-2021-318992 33741582 PMC8449789

[B12] Chithrapathra K. E. Hewanayake W. S. Egodage S. Silva S. (2023). Diabetic striatopathy: a case report of a patient with poor glycaemic control and abnormal movements. Cureus 15:e45581. doi: 10.7759/cureus.45581 37868561 PMC10587642

[B13] Chong D. D. Das N. Singh R. P. (2024). Diabetic retinopathy: screening, prevention, and treatment. Cleve. Clin. J. Med. 91, 503–510. doi: 10.3949/ccjm.91a.24028 39089852

[B14] Cole D. M. Smith S. M. Beckmann C. F. (2010). Advances and pitfalls in the analysis and interpretation of resting-state FMRI data. Front. Syst. Neurosci. 4:8. doi: 10.3389/fnsys.2010.00008 20407579 PMC2854531

[B15] Dai P. Yu Y. Sun Q. Yang Y. Hu B. Xie H. . (2024). Abnormal changes of brain function and structure in patients with T2DM-related cognitive impairment: a neuroimaging meta-analysis and an independent validation. Nutr. Diabetes 14:91. doi: 10.1038/s41387-024-00348-5 39528442 PMC11554684

[B16] De Ridder D. Vanneste S. Smith M. Adhia D. (2022). Pain and the triple network model. Front. Neurol. 13:757241. doi: 10.3389/fneur.2022.757241 35321511 PMC8934778

[B17] Duarte-Silva E. de Melo M. G. Maes M. Filho A. J. M. C. Macedo D. Peixoto C. A. (2021). Shared metabolic and neuroimmune mechanisms underlying type 2 diabetes mellitus and major depressive disorder. Prog. Neuropsychopharmacol. Biol. Psychiatry 111:110351. doi: 10.1016/j.pnpbp.2021.110351 34000290

[B18] Friedman E. (1994). CSPT circuitry in affective disorders. Biol. Psychiatry 36, 208–209. doi: 10.1016/0006-3223(94)91231-9 7948462

[B19] Fu Y. Gu M. Wang R. Xu J. Sun S. Zhang H. . (2023). Abnormal functional connectivity of the frontostriatal circuits in type 2 diabetes mellitus. Front. Aging Neurosci. 14:1055172. doi: 10.3389/fnagi.2022.1055172 36688158 PMC9846649

[B20] Ghamdi A. H. A. (2020). Clinical predictors of diabetic retinopathy progression; a systematic review. Curr. Diabetes Rev. 16, 242–247. doi: 10.2174/1573399815666190215120435 30767747

[B21] Hendrick A. M. Gibson M. V. Kulshreshtha A. (2015). Diabetic retinopathy. Prim. Care 42, 451–464. doi: 10.1016/j.pop.2015.05.005 26319349

[B22] Hörtnagl H. Pifl C. Hörtnagl E. Reiner A. Sperk G. (2020). Distinct gradients of various neurotransmitter markers in caudate nucleus and putamen of the human brain. J. Neurochem. 152, 650–662. doi: 10.1111/jnc.14897 31608979 PMC7078952

[B23] Hsu C. L. Manor B. Travison T. Pascual-Leone A. Lipsitz L. A. (2023). Sensorimotor and frontoparietal network connectivity are associated with subsequent maintenance of gait speed and episodic memory in older adults. J. Gerontol. Biol. Sci. Med. Sci. 78, 521–526. doi: 10.1093/gerona/glac193 36124711 PMC9977250

[B24] Huang X. Dan H. D. Zhou F. Q. Deng Q. Q. Shen Y. (2019). Abnormal intrinsic functional network hubs and connectivity following peripheral visual loss because of inherited retinal degeneration. Neuroreport 30, 295–304. doi: 10.1097/WNR.0000000000001200 30763285

[B25] Karnath H. O. Himmelbach M. Rorden C. (2002). The subcortical anatomy of human spatial neglect: putamen, caudate nucleus and pulvinar. Brain 125(Pt 2), 350–360. doi: 10.1093/brain/awf032 11844735

[B26] Kim K. S. Hong S. Han K. Park C. Y. (2024). Association of non-alcoholic fatty liver disease with cardiovascular disease and all cause death in patients with type 2 diabetes mellitus: nationwide population based study. BMJ. 384:e076388. doi: 10.1136/bmj-2023-076388 38350680 PMC10862140

[B27] Kohn N. Toygar T. Weidenfeld C. Berthold-Losleben M. Chechko N. Orfanos S. . (2015). In a sweet mood? Effects of experimental modulation of blood glucose levels on mood-induction during fMRI. Neuroimage 113, 246–256. doi: 10.1016/j.neuroimage.2015.03.024 25795339

[B28] Kour V. Swain J. Singh J. Singh H. Kour H. A. (2024). Review on diabetic retinopathy. Curr. Diabetes Rev. 20:e201023222418. doi: 10.2174/0115733998253672231011161400 37867267

[B29] Kroemer N. B. Opel N. Teckentrup V. Li M. Grotegerd D. Meinert S. . (2022). Functional connectivity of the nucleus accumbens and changes in appetite in patients with depression. JAMA Psychiatry 79, 993–1003. doi: 10.1001/jamapsychiatry.2022.2464 36001327 PMC9403857

[B30] Kwok C. S. Phillips A. Mukherjee S. Patel M. G. Hanif W. (2024). Missed opportunities in type 2 diabetes mellitus: a narrative review. Curr. Diabetes Rev. 20:e150124225648. doi: 10.2174/0115733998274651231117101511 38243953

[B31] Li S. Li P. Gong H. Jiang F. Liu D. Cai F. . (2017). Intrinsic functional connectivity alterations of the primary visual cortex in primary angle-closure glaucoma patients before and after surgery: a resting-state fMRI study. PLoS ONE 12:e0170598. doi: 10.1371/journal.pone.0170598 28122025 PMC5266295

[B32] Liang L. Fratzl A. Goldey G. Ramesh R. N. Sugden A. U. Morgan J. L. . (2018). A fine-scale functional logic to convergence from retina to Thalamus. Cell 173, 1343–1355.e24. doi: 10.1016/j.cell.2018.04.041 29856953 PMC6003778

[B33] Liu J. Liu T. Wang W. Ma L. Ma X. Shi S. . (2017). Reduced gray matter volume in patients with type 2 diabetes mellitus. Front. Aging Neurosci. 9:161. doi: 10.3389/fnagi.2017.00161 28588480 PMC5439076

[B34] Liu X. Chen L. Cheng R. Luo T. Lv F. Fang W. . (2019). Altered functional connectivity in patients with subcortical ischemic vascular disease: a resting-state fMRI study. Brain Res. 1715, 126–133. doi: 10.1016/j.brainres.2019.03.022 30910630

[B35] Meng J. Liu J. Li H. Gao Y. Cao L. He Y. . (2022). Impairments in intrinsic functional networks in type 2 diabetes: a meta-analysis of resting-state functional connectivity. Front. Neuroendocrinol. 66:100992. doi: 10.1016/j.yfrne.2022.100992 35278579

[B36] Nilofar F. Ganapathy G. Bose S. Unraveling Diabetic Striatopathy V. V. (2024). Clinical and imaging perspectives. Cureus 16:e67105. doi: 10.7759/cureus.67105 39290934 PMC11407699

[B37] Peters A. J. Fabre J. M. J. Steinmetz N. A. Harris K. D. Carandini M. (2021). Striatal activity topographically reflects cortical activity. Nature 591, 420–425. doi: 10.1038/s41586-020-03166-8 33473213 PMC7612253

[B38] Pritz M. B. (2025). Thalamus of reptiles and mammals: some significant differences. Brain Behav. Evol. 100, 49–57. doi: 10.1159/000542100 39427637

[B39] Qi C. X. Huang X. Tong Y. Shen Y. (2021). Altered functional connectivity strength of primary visual cortex in subjects with diabetic retinopathy. Diabetes Metab. Syndr. Obes. 14, 3209–3219. doi: 10.2147/DMSO.S311009 34285528 PMC8286104

[B40] Rolls E. T. Cheng W. Feng J. (2020). The orbitofrontal cortex: reward, emotion and depression. Brain Commun. 2:fcaa196. doi: 10.1093/braincomms/fcaa196 33364600 PMC7749795

[B41] Schreiner D. C. Gremel C. M. (2018). Orbital frontal cortex projections to secondary motor cortex mediate exploitation of learned rules. Sci. Rep. 8:10979. doi: 10.1038/s41598-018-29285-x 30030509 PMC6054681

[B42] Sendi M. S. E. Zendehrouh E. Fu Z. Liu J. Du Y. Mormino E. . (2023). Disrupted dynamic functional network connectivity among cognitive control networks in the progression of Alzheimer's disease. Brain Connect. 13, 334–343. doi: 10.1089/brain.2020.0847 34102870 PMC10442683

[B43] Skalkos S. Moschonis G. Thomas C. J. McMillan J. Kouris-Blazos A. (2020). Effect of lupin-enriched biscuits as substitute mid-meal snacks on post-prandial interstitial glucose excursions in post-surgical hospital patients with type 2 diabetes. Nutrients 12:1239. doi: 10.3390/nu12051239 32349429 PMC7281993

[B44] Strati M. Moustaki M. Psaltopoulou T. Vryonidou A. Paschou S. A. (2024). Early onset type 2 diabetes mellitus: an update. Endocrine 85, 965–978. doi: 10.1007/s12020-024-03772-w 38472622 PMC11316703

[B45] Sun D. M. Ma Y. Sun Z. B. Xie L. Huang J. Z. Chen W. S. . (2017). Decision-making in primary onset middle-age type 2 diabetes mellitus: a BOLD-fMRI study. Sci. Rep. 7:10246. doi: 10.1038/s41598-017-10228-x 28860463 PMC5579021

[B46] Sun J. Xu L. Ma Y. Guo C. Du Z. Gao S. . (2023). Different characteristics of striatal resting-state functional conectivity in treatment-resistant and non-treatment-resistant depression. Psychiatry Res. Neuroimaging 328:111567. doi: 10.1016/j.pscychresns.2022.111567 36462466

[B47] Tatsumi T. (2023). Current treatments for diabetic macular edema. Int. J. Mol. Sci. 24:9591. doi: 10.3390/ijms24119591 37298544 PMC10253534

[B48] Volkow N. D. Wang G. J. Fowler J. S. Tomasi D. Telang F. (2011). Addiction: beyond dopamine reward circuitry. Proc. Natl. Acad. Sci. U.S.A. 108, 15037–15042. doi: 10.1073/pnas.1010654108 21402948 PMC3174598

[B49] Wagner F. Rogenz J. Opitz L. Maas J. Schmidt A. Brodoehl S. . (2023). Reward network dysfunction is associated with cognitive impairment after stroke. Neuroimage Clin. 39:103446. doi: 10.1016/j.nicl.2023.103446 37307650 PMC10276182

[B50] Wang L. Li F. Mitchell P. B. Wang C. Y. Si T. M. (2020). Striatal resting-state connectivity abnormalities associated with different clinical stages of major depressive disorder. J. Clin. Psychiatry 81:19m12790. doi: 10.4088/JCP.19m12790 32078260

[B51] Wang L. Wang K. Liu J. H. Wang Y. P. (2018). Altered default mode and sensorimotor network connectivity with striatal subregions in primary insomnia: a resting-state multi-band fMRI study. Front. Neurosci. 12:917. doi: 10.3389/fnins.2018.00917 30574065 PMC6291517

[B52] Wang X. Luo P. Zhang L. Sun J. Cao J. Lei Z. . (2024). Altered functional brain activity in first-episode major depressive disorder treated with electro-acupuncture: a resting-state functional magnetic resonance imaging study. Heliyon 10:e29613. doi: 10.1016/j.heliyon.2024.e29613 38681626 PMC11053281

[B53] Wang Z. L. Zou L. Lu Z. W. Xie X. Q. Jia Z. Z. Pan C. J. . (2017). Abnormal spontaneous brain activity in type 2 diabetic retinopathy revealed by amplitude of low-frequency fluctuations: a resting-state fMRI study. Clin. Radiol. 72, 340.e1–340.e7. doi: 10.1016/j.crad.2016.11.012 28041652

[B54] Wen Z. Zhou F. Q. Huang X. Dan H. D. Xie B. J. Shen Y. (2018). Altered functional connectivity of primary visual cortex in late blindness. Neuropsychiatr. Dis. Treat. 14, 3317–3327. doi: 10.2147/NDT.S183751 30584305 PMC6284854

[B55] Wong T. Y. Klein R. Islam F. M. Cotch M. F. Folsom A. R. Klein B. E. . (2006). Diabetic retinopathy in a multi-ethnic cohort in the United States. Am. J. Ophthalmol. 141, 446–455. doi: 10.1016/j.ajo.2005.08.063 16490489 PMC2246042

[B56] Yan W. Limin G. Zhizhong S. Zidong C. Shijun Q. (2025). Altered individual-based morphological brain network in type 2 diabetes mellitus. Brain Res. Bull. 222:111228. doi: 10.1016/j.brainresbull.2025.111228 39892582

[B57] Yan W. Wang Y. (2023). Clinical study of Chinese medicine holographic scraping combined with hot ironing in improving early diabetic retinopathy. Am. J. Transl. Res. 15, 511–521. 36777822 PMC9908488

[B58] Yao L. Yang C. Zhang W. Li S. Li Q. Chen L. . (2021). A multimodal meta-analysis of regional structural and functional brain alterations in type 2 diabetes. Front. Neuroendocrinol. 62:100915. doi: 10.1016/j.yfrne.2021.100915 33862036

[B59] Yuan Y. Dong M. Wen S. Yuan X. Zhou L. (2024). Retinal microcirculation: a window into systemic circulation and metabolic disease. Exp. Eye Res. 242:109885. doi: 10.1016/j.exer.2024.109885 38574944

[B60] Yue T. Shi Y. Luo S. Weng J. Wu Y. Zheng X. (2022). The role of inflammation in immune system of diabetic retinopathy: molecular mechanisms, pathogenetic role and therapeutic implications. Front. Immunol. 13:1055087. doi: 10.3389/fimmu.2022.1055087 36582230 PMC9792618

[B61] Zhang D. Wang M. Gao J. Huang Y. Qi F. Lei Y. . (2021). Altered functional connectivity of insular subregions in type 2 diabetes mellitus. Front. Neurosci. 15:676624. doi: 10.3389/fnins.2021.676624 34220433 PMC8242202

[B62] Zhang X. Chen Z. Li Y. Xie C. Liu Z. Wu Q. . (2024). Volume development changes in the occipital lobe gyrus assessed by MRI in fetuses with isolated ventriculomegaly correlate with neurological development in infancy and early childhood. J. Perinatol. 44, 1178–1185. doi: 10.1038/s41372-024-02012-3 38802655

